# Pica, a complex differential diagnosis. A case report

**DOI:** 10.1192/j.eurpsy.2025.1407

**Published:** 2025-08-26

**Authors:** I. Ramos Suárez, M. Guerrero Jiménez

**Affiliations:** 1 Psychiatry, San Cecilio University Hospital; 2 Psychiatry, Granada University of Medicine, Granada, Spain

## Abstract

**Introduction:**

The Diagnostic and Statistical Manual of Mental Disorders, Fifth Edition (DSM-5) defines pica as the ingestion of non-nutritive and non-food substances. For a diagnosis to be made, the behavior must persist for at least one month, not be consistent with the child’s developmental stage, and not be a socially normative or culturally accepted practice. The etiology of pica is not well understood. Additionally, knowledge about its overall prevalence is limited.

**Objectives:**

We present the case of a 61-year-old female patient diagnosed with histrionic personality disorder and generalized anxiety disorder, who began to frequently visit the hospital emergency department due to the ingestion of various objects, such as screws, paper clips… (Image 1). It was decided to admit her to the psychiatric ward with two main objectives:To halt the progression of disruptive behaviors and to determine the underlying cause of the pica behaviors.

**Methods:**

During hospitalization, no clear affective disturbances toward any polarity were observed. Additionally, the patient was unable to coherently justify her behaviors, merely describing them as “spontaneous and irresistible impulses.” However, significant cognitive impairment was evident during the admission. Furthermore, the presence of balance disturbances with lower limb incoordination and urinary incontinence was confirmed. The described triad—gait disturbances, urinary incontinence, and cognitive deterioration—is characteristic of adult chronic hydrocephalus (ACH). Given this suspicion, a cranial computed tomography (CT) scan, was requested.

**Results:**

The Evans’ index (EI) was calculated. An EI greater than 0.30 is indicative of ventricular dilation, and the patient’s EI was 0.39 (Image 2). The high clinical suspicion, insidious onset, and the ventricular dilation observed made the diagnosis of ACH highly probable. Consequently, the patient was referred to the neurosurgery department to evaluate the potential placement of a cerebrospinal fluid shunt.

**Image 1:**

**Figure:**
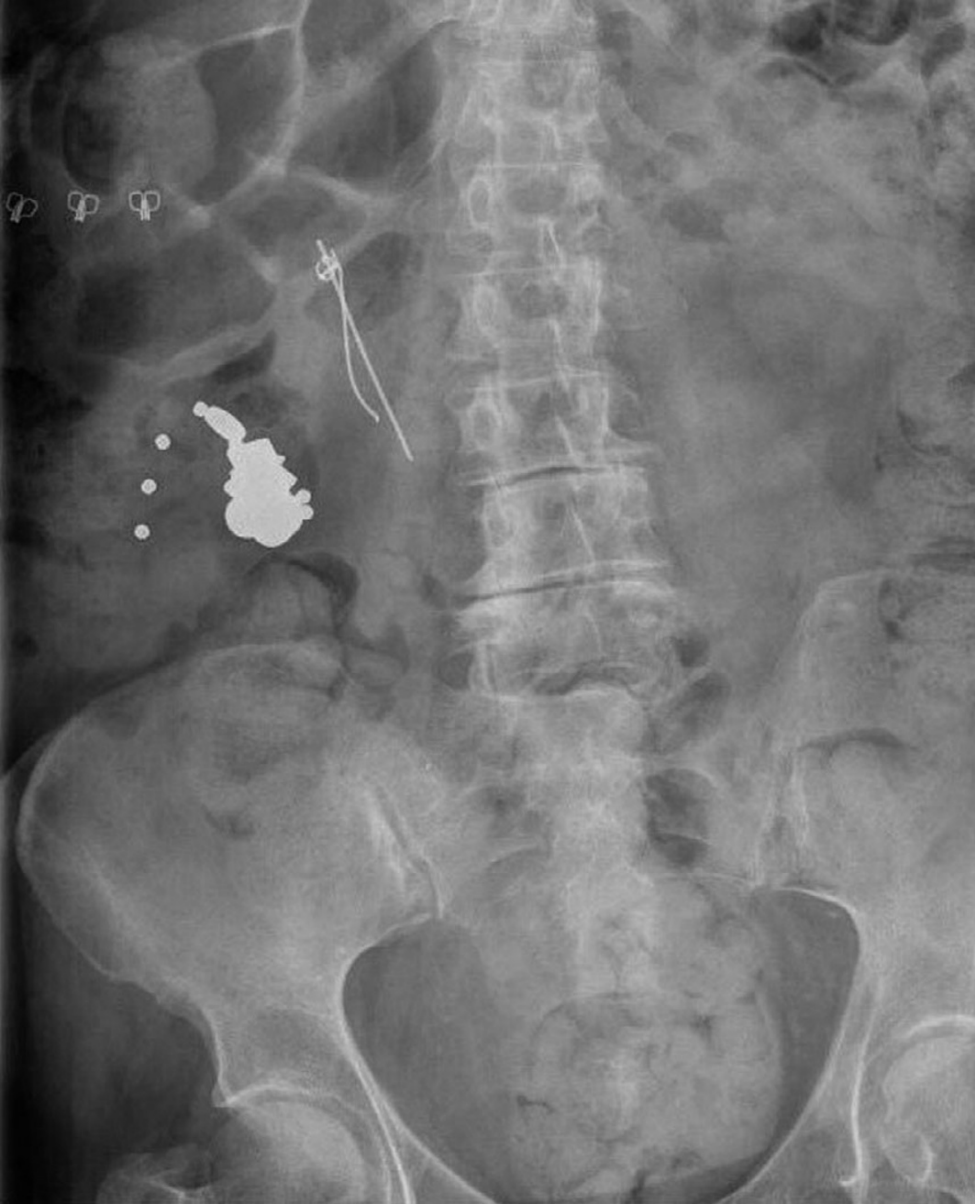

**Image 2:**

**Figure:**
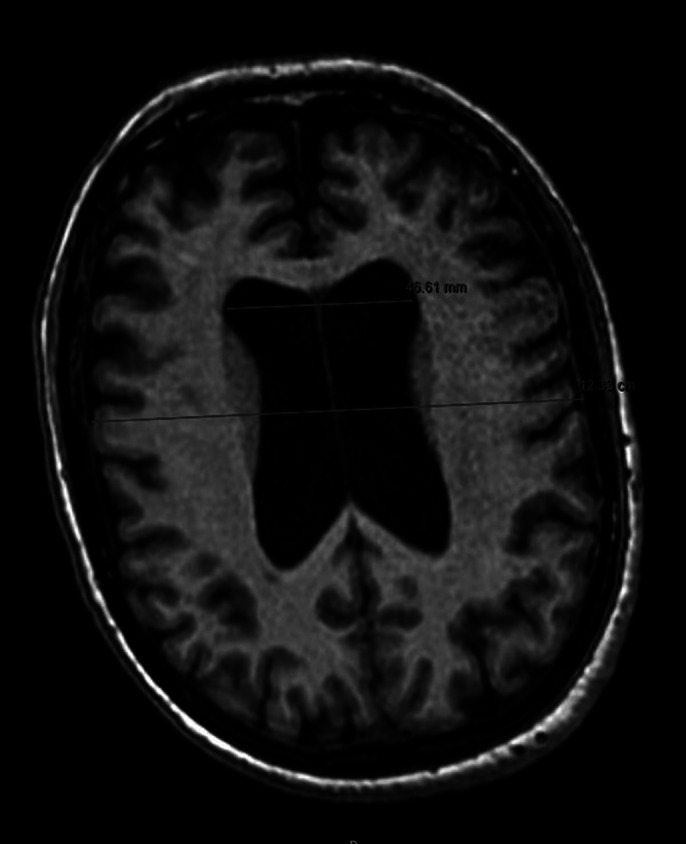

**Conclusions:**

Although pica behaviors can be associated with various psychiatric disorders, there is often an underlying organic substrate. In this patient, the initial diagnosis of personality disorders and generalized anxiety, coupled with the repetitive pica behaviors, might have initially pointed exclusively toward a psychiatric approach. The identification of a broader symptomatic pattern, led to the suspicion of adult chronic hydrocephalus (ACH). The triad of symptoms in ACH, though classic, may not always be evident, and neuropsychiatric manifestations such as pica could be indirect signs of brain involvement.

This case underscores the need to consider organic differential diagnoses in patients presenting with atypical or unexplained behaviors from a purely psychiatric standpoint.

**Disclosure of Interest:**

None Declared

